# BMA-Net: A Bilateral Attention Network with Retinal Domain Transfer-Learning for CIMT-Based Cardiovascular Risk Classification from Fundus Images

**DOI:** 10.3390/bioengineering13101168

**Published:** 2026-10-08

**Authors:** Mingze Lyu, Abhirup Banerjee

**Affiliations:** Institute of Biomedical Engineering, Department of Engineering Science, University of Oxford, Oxford OX3 7DQ, UK; mingze.lyu@mansfield.ox.ac.uk

**Keywords:** carotid intima-media thickness, retinal fundus imaging, convolutional neural network, attention mechanism, bilateral image fusion

## Abstract

Carotid intima-media thickness (CIMT) is an established marker of subclinical atherosclerosis, but its reliance on ultrasound equipment and trained operators limits its suitability for large-scale screening. Retinal fundus photography may offer a more scalable approach to vascular assessment, as it contains retinal vascular features associated with systemic cardiovascular health and can be acquired quickly and non-invasively. This study proposes a two-stage transfer learning framework for CIMT-based cardiovascular risk classification. An attention-enhanced EfficientNet-B4 is first pretrained on the Asia Pacific Tele-Ophthalmology Society 2019 Blindness Detection dataset and subsequently transferred to a weight-sharing Siamese architecture to classify the China Fundus Carotid Intima-Media Thickness dataset. Across ten independent initializations, the proposed model achieved an average macro-F1 score of 77.81% and an area under the receiver operating characteristic curve of 84.08% on the validation set and 78.29% and 85.22%, respectively, on the test set. It achieved overall higher point estimates for performance metrics than those previously reported for the Siamese ResNeXt baseline model with squeeze-and-excitation attention, and demonstrated a reduced disparity in class-wise performance. Grad-CAM analysis further revealed differences in the spatial activation patterns between models trained under different experimental configurations. Overall, these findings support the feasibility of fundus imaging as a scalable and non-invasive approach to CIMT-based cardiovascular risk assessment.

## 1. Introduction

Cardiovascular diseases (CVDs) remain a major cause of morbidity and mortality worldwide [1]. Atherosclerosis, characterized by progressive changes in the arterial wall, is an important contributor to cardiovascular disease and can eventually result in clinical events such as myocardial infarction and ischemic stroke [2]. In particular, atherosclerotic vascular changes can develop over many years before overt cardiovascular symptoms occur. Therefore, identifying subclinical vascular changes before major cardiovascular events is essential for early detection of atherosclerotic changes and cardiovascular risk [3].

Carotid intima-media thickness (CIMT), measured non-invasively via carotid ultrasonography, provides meaningful information about the arterial wall structure. As a marker of subclinical vascular disease, increased CIMT is associated with higher risk of cardiovascular events. Specifically, Lorenz et al. [4] reported that a 0.1 mm increment in CIMT is associated with a 10–15% increase in myocardial infarction risk and a 13–18% increase in stroke risk after adjusting for age and sex. Moreover, CIMT provides a fundamentally different type of information from the conventional risk factors such as age, smoking status, blood pressure, or serum lipids. These are upstream physiological conditions that are hypothesized to promote atherosclerosis, while CIMT is a direct structural readout of atherosclerotic changes in the arterial wall that have developed over time. Therefore, CIMT is able to capture aspects of cumulative cardiovascular risk exposure that are not fully represented by risk factors measured at a single time point [5]. However, despite its various advantages, CIMT is not routinely measured in clinical practice, as its assessment requires dedicated equipment and trained operators, making it less readily available than the conventional risk factor measurements [6]. It is also important to note that the CIMT-based cardiovascular risk should not be considered equivalent to clinical cardiovascular risk prediction based on validated risk scores, which incorporates a more comprehensive range of clinical risk factors.

In this context, retinal fundus photography offers an attractive alternative for CIMT-based cardiovascular risk prediction. First, fundus photography is already well-established in routine ophthalmic screening, which makes retinal images comparatively cheap and easy to collect at scale [7]. More importantly, in the human body, the retina is the only site where the microvasculature can be viewed directly and non-invasively. Since it is exposed to many of the same systemic haemodynamic and metabolic influences as the rest of the vascular system, retinal vessel caliber, tortuosity, and branching geometry have long been associated with systemic cardiovascular conditions [8,9]. Recent studies have also demonstrated important associations between retinal microvascular alterations and carotid stenosis, while specific retinal vascular metrics have also been shown to directly correlate with CIMT [10,11].

In particular, the growing prevalence of deep learning in extracting cardiovascular risk information from retinal fundus images provides substantial motivation for extending this approach to CIMT-based cardiovascular risk prediction [12,13,14]. This relationship was first explored by Gong et al. [15] in a subpopulation of patients with type 2 diabetes mellitus (T2DM), where the model achieved relatively strong macro-F1 scores of 88.00% and 85.00% on the validation and test sets respectively. When they extended their work to the more heterogeneous general population in [16], the performance declined substantially with the reported validation and test macro-F1 being 77.20% and 74.94% respectively. This suggests that the association between retinal characteristics and CIMT-based cardiovascular risk becomes considerably more challenging to learn as population heterogeneity increases.

To better understand the limitations in their work that might affect model performance, we identify two key factors that provide potential avenues for improvement. On the one hand, the limited availability of relevant training data represents an important constraint. In this regard, the China Fundus Carotid Intima-Media Thickness (China-Fundus-CIMT) dataset [16] contains bilateral fundus photographs from 2903 participants, which remains modest in scale. On the other hand, model initialization may further limit its generalizing capability. Both studies initialize their models using ImageNet weights [15,16], which provide generic visual representations learned from natural images. However, natural images differ substantially from retinal fundus in terms of appearance, acquisition characteristics, anatomical content, and clinically relevant structures, creating a domain mismatch that might constrain the effectiveness of transfer learning to highly specialized tasks [17,18].

**Figure 1:**
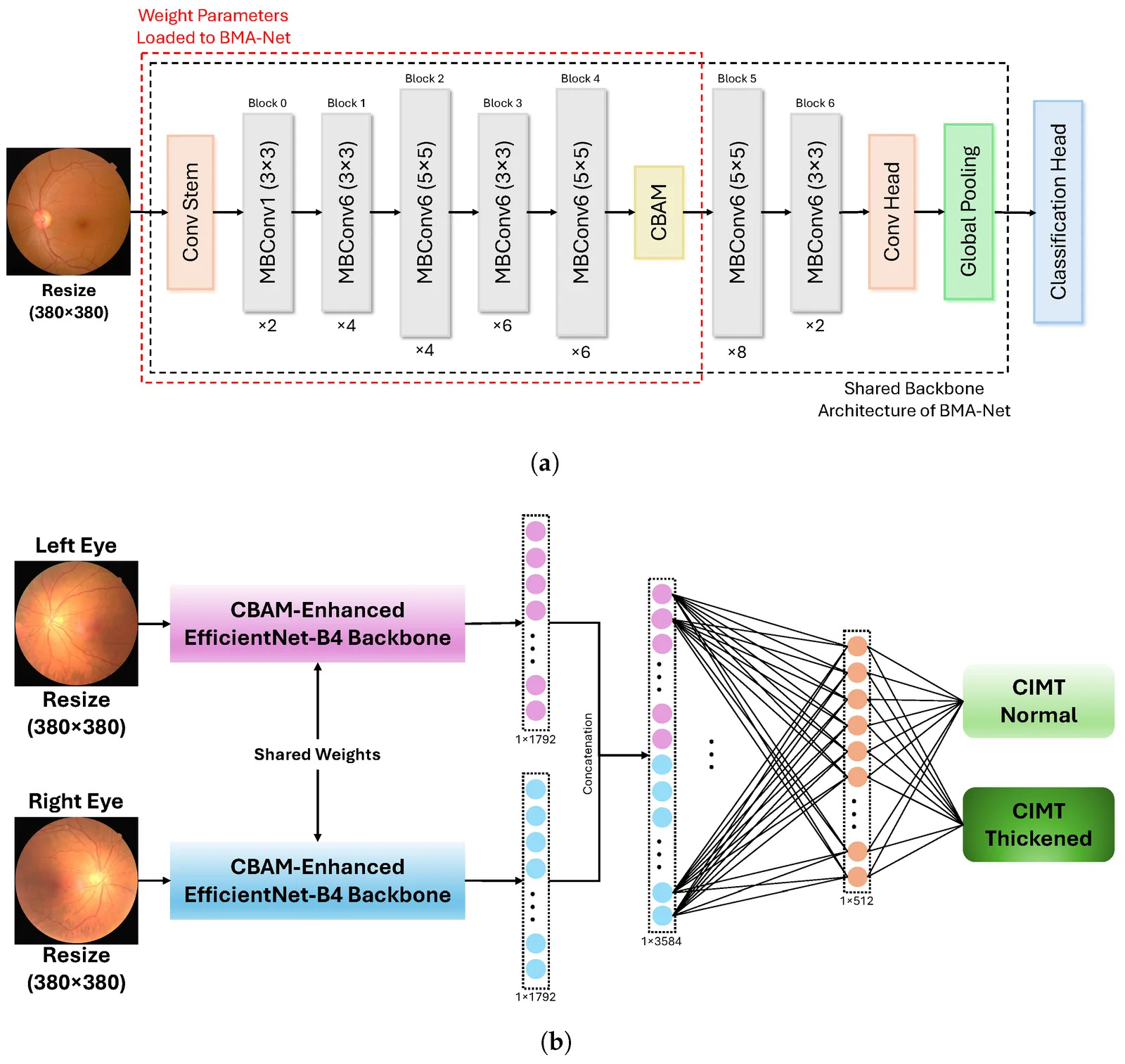
Architecture of the proposed BMA-Net framework. (**a**) CBAM-enhanced EfficientNet-B4 backbone used for retinal domain pretraining on APTOS-2019. CBAM is inserted between blocks 4 and 5, and the backbone components within the dashed box are subsequently transferred to initialize BMA-Net, while the diabetic retinopathy classification head is discarded. (**b**) Bilateral MBConv attention network (BMA-Net) for CIMT-based cardiovascular risk classification. The left- and right-eye fundus images are processed by weight-sharing branches initialized from the pretrained backbone. The resulting 1792-dimensional feature representations are concatenated and passed through an MLP to produce the final classification.

To obtain a more domain-relevant initialization than ImageNet, we introduce an intermediate pretraining stage using the Asia Pacific Tele-Ophthalmology Society 2019 Blindness Detection (APTOS-2019) dataset [19]. We first initialize an EfficientNet-B4 backbone [20] with a Convolutional Block Attention Module (CBAM) [21] (Figure 1a) and ImageNet weights and train it for diabetic retinopathy (DR) gradings on APTOS-2019. Although DR gradings differ from CIMT-based cardiovascular risk classification, this stage exposes the model to retina-specific structures that may generalize better to the downstream task. The pretrained backbone is then transferred to both branches of the Bilateral MBConv Attention Network (BMA-Net) (Figure 1b). The two branches share the same weights to process the bilateral fundus independently with a common feature extractor. During fine-tuning, progressive unfreezing and discriminative learning rates are used. We also conduct ablation studies to examine the individual effects of proposed pretraining and fine-tuning strategies. To account for the training stochasticity, all experiments are repeated across ten independently seeded runs, and each setting is evaluated using both its average performance and variability across runs. Moreover, Grad-CAM [22] is used to examine the spatial regions contributing to the model predictions.

The main contributions of this study are summarized as follows:

- We propose a two-stage transfer learning framework that introduces retinal domain pretraining on APTOS-2019 before adaptation to CIMT-based cardiovascular risk classification, providing a more domain relevant initialization.
- We develop BMA-Net, a weight-sharing bilateral architecture based on CBAM-enhanced EfficientNet-B4, which independently extracts features from the left and right fundus images and integrates them for subject-level classification.
- We employ progressive unfreezing with discriminative learning rates to preserve transferable retinal representations while gradually adapting the network to CIMT-based cardiovascular risk classification task.
- We evaluate the proposed framework across ten independent training runs and complementary ablations, demonstrating higher classification metrics and reduced class-wise disparity.

## 2. Methods

### 2.1. Dataset

#### 2.1.1. China-Fundus-CIMT

The China Fundus Carotid Intima-Media Thickness (China-Fundus-CIMT) dataset enables modelling of the relationship between readily obtainable retinal fundus images and CIMT values under a supervised learning framework, where CIMT provides a quantitative indicator of carotid vascular condition and CIMT-based cardiovascular risk [16]. In particular, CIMT measurements were captured using a Siemens ACUSON S2000 ultrasound diagnostic system equipped with an L16 probe operating at 5–12 MHz. Measurements were obtained from the distal wall of the common carotid artery, 1–2 cm proximal to the carotid bifurcation, while the corresponding fundus photographs were acquired using a Canon CR-2 PLUS AF non-mydriatic digital fundus camera. All subjects in the dataset had undergone both examinations during hospitalization [16].

The dataset comprises 2903 subjects, each associated with a pair of bilateral fundus images. The measured CIMT values were retained in millimeters and were directly used to define the two diagnostic groups: subjects with CIMT < 0.9 mm were classified as normal, whereas those with CIMT ≥ 0.9 mm were classified as thickened [16]. As a result, 849 cases were annotated as normal and 2054 cases as CIMT thickened, reflecting a notable class imbalance. The dataset was further partitioned into independent training, validation, and test sets. The training set contains 699 normal and 1904 thickened subjects, while the validation and test sets include 100 normal and 100 thickened subjects, and 50 normal and 50 thickened subjects, respectively. All cases are accompanied by structured metadata, including patient IDs, diagnostic labels, and auxiliary clinical information, stored in JSON format to facilitate efficient indexing and data loading.

#### 2.1.2. APTOS-2019

The Asia Pacific Tele-Ophthalmology Society 2019 Blindness Detection (APTOS-2019) dataset comprises 5590 retinal fundus images collected and organized by the Aravind Eye Hospital in India [19]. Among which, 3662 constitute the dataset used in this study. These subjects were annotated according to the International Clinical Diabetic Retinopathy Disease Severity Scale (ICDRSS), which defines five categories: no Diabetic Retinopathy (DR), mild DR, moderate DR, severe DR, and proliferative DR. Following the sample split provided in a Kaggle release [23], these images were further divided into training, validation and test sets, each with 2930, 366, and 366 subjects, respectively. As the explicit patient identifiers are unavailable, the predefined dataset partitions and labels were retained for this study.

This dataset provides a diverse representation of pathological retinal features across different levels of DR severity. Learning to distinguish these severity levels requires the model to capture variations in retinal vascular and morphological characteristics associated with DR progression. Such representations may be particularly relevant to the downstream task, as associations between DR severity and increased CIMT have been reported previously [24,25]. Although features underlying the two conditions are not necessarily identical, their shared underlying fundus-specific features including vascular patterns, optic disc morphology, background texture, contrast, and variations in illumination provide a rationale for using DR classification as an intermediate pretraining task before adaptation to CIMT-based cardiovascular risk classification.

To ensure the generalizability of learned retinal representations and avoid over-parameterization toward DR-specific markers, we only aim to achieve a reliable separation between healthy and pathological cases. However, rather than collapsing the four DR severity levels into a single category, we retain the original class labels to preserve subtle morphological information and encourage the model to encode rich features that are transferable to the downstream task.

### 2.2. Transfer Learning Pipeline

The proposed transfer learning pipeline consists of two sequential stages: pretraining in the retinal domain with the APTOS-2019 dataset and task-specific fine-tuning with the China-Fundus-CIMT dataset.

For the first stage of pretraining in the retinal domain, we propose an EfficientNet-B4 architecture [20] integrated with the Convolutional Block Attention Module (CBAM) [21] and a task-specific classification head for DR grading, as illustrated in Figure 1a. The architecture is first initialized with ImageNet weights [26] to capture the generic visual patterns. Subsequently, the model is trained in a supervised manner on the APTOS-2019 dataset to capture the domain-specific features like retinal vessel morphology, optic disc boundaries, and retinal contrast variations.

Following pretraining in the retinal domain, the learned convolutional feature extractor is transferred to the proposed Bilateral MBConv Attention Network (BMA-Net) in Figure 1b for further optimization. Specifically, the architectures enclosed by the dashed box in Figure 1a, including the EfficientNet blocks, inserted CBAM module, and convolutional head, are used to initialize two identical branches of the BMA-Net, whereas the task-specific classification head for DR grading is discarded.

During fine-tuning, the CBAM module and all its preceding layers are initially frozen to preserve the low and middle level retinal representations acquired from retinal domain pretraining. The entire network is subsequently optimized with the training strategy of progressive unfreezing with discriminative learning rates to facilitate stable adaptation of the learned representations. The detailed initialization and training schemes are elaborated in Section 2.7.

### 2.3. Model Architecture Design

Our aim in this study is to infer CIMT-based cardiovascular risk from retinal fundus images, while characterizing retinal features most informative for CIMT-defined cardiovascular risk [15], with the CIMT values serving as the clinical reference for the definition of risk labels of 0 and 1 [16]. This motivates the use of data-driven representation learning to directly identify useful discriminative features and semantics from fundus images. As these learned representations are not inherently interpretable, attention mechanisms are incorporated to encourage the model to prioritize informative feature channels and spatial regions of the retinal fundus [27].

#### 2.3.1. Strategy of Bilateral Fundus Integration

A key advantage of the China-Fundus-CIMT dataset is the availability of bilateral fundus images. Therefore, the model architecture is carefully designed to effectively integrate the complementary retinal information in the bilateral images pair. Previous literature investigated this problem by evaluating and comparing three strategies: Image Stitching, Parallel Learning, and Siamese Learning [15]. Among these configurations, the Siamese Learning architecture with the dual weight sharing branches consistently achieved superior classification performance. Given the same bilateral input setting and the common objective of classifying subjects according to CIMT-defined cardiovascular risk, the Siamese Learning paradigm is therefore retained as the foundation of the proposed architecture.

#### 2.3.2. Backbone and Attention Module Selection

Selection of an appropriate feature extraction backbone for the Siamese configuration governs the network’s representational capacity and computational efficiency [28]. Previous convolutional neural network (CNN) architectures have improved representation learning through several complementary dimensions including network depth, width, cardinality, and attention. The original study [16] adopted ResNeXt [29] for the backbone, which extends the residual learning framework by introducing cardinality through grouped convolutions, thereby increasing representational capacity without requiring a proportional increase in computational complexity. A Squeeze-and-Excitation (SE) network [30] was also introduced to recalibrate the channel-wise feature responses. The resulting Siamese SE-ResNeXt architecture therefore integrates cardinality-based representation learning with channel-wise feature recalibration, which establishes a useful architectural baseline for further modification.

Building upon the baseline, we retain the Siamese Learning configuration, while the ResNeXt backbone is replaced with EfficientNet-B4 [20], which jointly scales network depth, width, and input resolution using the compound scaling strategy. Its Mobile Inverted Bottleneck Convolution (MBConv) blocks provide an efficient mechanism for feature extraction through inverted residual connections and depthwise separable convolutions. This design shifts the emphasis from increasing representational capacity through cardinality, as adopted by ResNeXt, towards a more systematic balance between model capacity and computational cost.

For the attention module, although the SE mechanism adopted by Guo et al. [16] can strengthen responses from informative feature channels, it operates exclusively along the channel dimension and does not explicitly model spatial importance. Other common attention mechanisms like the Efficient Channel Attention (ECA) [31] similarly focus on channel-wise feature interactions, whereas the Bottleneck Attention Module (BAM) [32] and Convolutional Block Attention Module (CBAM) [21] additionally enable spatial feature refinement. In this study, CBAM is incorporated into the EfficientNet-B4 backbone, as illustrated in Figure 1a, due to its sequential channel and spatial attention mechanisms and lightweight nature. This design is particularly meaningful for fundus image analysis, as the discriminative information for classification might depend on both the importance of individual feature channels and the spatial focus of retinal features.

#### 2.3.3. Rationale of CBAM Placement

Although clinical studies have reported an association between increasing DR severity and higher CIMT, this association does not necessarily imply that the same retinal regions and feature responses would be informative for both classification tasks. In CNN architectures, shallow layers generally capture low-level and generic features, whereas deeper layers progressively encode more task-specific representations [33]. Therefore, the shallow layers of the DR pretrained backbone are expected to retain a high degree of transferability to the CIMT-based classification task, particularly because both tasks operate within the same retinal fundus domain [34]. This transferability is expected to diminish with increasing network depth, as the learned representations become progressively specialized to retinal manifestations associated with DR.

Based on the above rationale, CBAM is inserted at an intermediate stage of the EfficientNet-B4 backbone, between blocks 4 and 5, as illustrated in Figure 1a. At this depth, retinal feature extractors are well developed to encode sufficiently discriminative patterns while remaining less specialized to the original DR classification task. CBAM therefore provides a mechanism for readjusting the relative importance of these intermediate feature responses according to their relevance to the downstream task of CIMT-based cardiovascular risk classification. Although CBAM is commonly placed after the final convolutional block, such a placement in the present transfer learning setting would restrict attention-based recalibration during transfer learning to the deepest and most abstract feature representations, which are more strongly conditioned by the DR classification task and less transferable to the CIMT-based task.

#### 2.3.4. Overall Architecture: BMA-Net

The resulting architecture, termed Bilateral MBConv Attention Network (BMA-Net), is shown in Figure 1b. Each fundus image is processed by one branch of the CBAM-enhanced EfficientNet-B4 backbone, with parameters shared across both branches. Following feature extraction, global average pooling transforms the output of each branch into a 1792-dimensional feature vector. The two feature vectors are then concatenated into a single 3584-dimensional feature vector of bilateral representation, which is subsequently passed through a multilayer perceptron (MLP). Within the MLP, the 3584-dimensional feature vector is first projected onto a 512-dimensional representation through a fully connected layer, followed by a ReLU activation. Another fully connected layer then maps this representation to the binary output classes for CIMT-based cardiovascular risk classification.

### 2.4. Data Sampling and Augmentation Strategy

In this work, we employ a soft balanced sampling strategy using PyTorch’s WeightedRandomSampler. For each class, the class weights are computed as

(1) $$w_{c}={\left(\frac{1}{n_{c}}\right)}^{1-\lambda }$$

where $n_{c}$ denotes the number of training samples belonging to class *c* and λ∈[0,1] is the softness parameter which modulates the degree of class balancing of the training data.

These class weights are subsequently propagated to individual samples in the dataset based on their respective class memberships, which are further normalized into probabilities by the WeightedRandomSampler. During training, samples are randomly drawn with replacement according to these probabilities. The total number of samples drawn per epoch is set to be equal to the size of the training set to maintain a constant epoch duration.

As a result, subjects from the minority class are sampled more frequently to yield a more balanced data distribution for the model during optimization. To mitigate the risk of overfitting inherently associated with the repeated sampling of minority class images, data augmentation is applied to the training pipeline. Firstly, all images for both training and validation are resized to 380×380 pixels to match the standard input resolution of the EfficientNet-B4 architecture. Training images are then dynamically augmented using random rotations ($\pm 10^{\circ }$) and color jittering (brightness ±20%, contrast ±20%, saturation ±20%, and hue ±0.03). Finally, all images are normalized using the standard ImageNet mean and standard deviation.

This data sampling framework supports a tunable softness hyperparameter λ; its optimal value is intrinsically dataset dependent, governed by factors such as the baseline class distribution and the specific nature of the imaging data. In this study, a baseline value of λ=0 is adopted for both pretraining and fine-tuning to maximize the model’s exposure to underrepresented minority class samples.

### 2.5. Loss Function

In this work, we employ focal loss [35] as the optimization objective for both pretraining and fine-tuning in the training pipeline. Compared to the conventional cross-entropy loss, focal loss introduces an additional modulating factor that suppresses the loss contribution by well-classified samples, enabling the model to focus on more challenging subjects during training.

While various formulations of focal loss exist in the literature, we employ the following variant:

(2) $$L_{\mathrm{FL}}=-\frac{1}{N}\sum_{i=1}^{N}\alpha_{t_{i}}{\left(1-p_{t_{i}}\right)}^{\gamma }\log\left(p_{t_{i}}\right)$$

where *N* denotes the number of training samples, $p_{t_{i}}$ represents the predicted probability of the ground truth class $t_{i}$, $\alpha_{t_{i}}$ is the class-specific weighting factor, and γ is the focusing parameter.

From the mathematical formulation, we can observe that assigning a larger weighting factor $\alpha_{t_{i}}$ to minority classes compensates for the class imbalance, while the focusing parameter suppresses the loss contribution by confidently predicted samples via the modulating factor ${\left(1-p_{t_{i}}\right)}^{\gamma }$. Specifically, for both pretraining on APTOS-2019 and fine-tuning on China-Fundus-CIMT, the class-specific weighting factor is computed based on the inverse class frequency. In particular, the inverse frequency class weights are smoothed by applying a square root transformation, followed by mean normalization [36]:

(3) $$\alpha_{c}=\frac{\frac{1}{\sqrt{n_{c}}}}{\frac{1}{K}\sum_{j=1}^{K}\frac{1}{\sqrt{n_{j}}}}$$

where $n_{c}$ denotes the number of training samples belonging to class *c*, and *K* is the total number of classes. Additionally, a focusing parameter of γ=2 is adopted for both stages, which is a well-established standard choice in many focal loss applications. This moderate weighting scheme is intentionally adopted to suppress the excessively large gradients from rare classes, which otherwise destabilize the training process.

The utility of the loss function is further demonstrated in the context of training data. The APTOS-2019 dataset exhibits severe class imbalance, with the “No DR” category constituting a large majority of easily classifiable training samples. Focal loss could mitigate this by shifting the optimization emphasis towards the minority and challenging samples, encouraging the model to extract more discriminative features for the underrepresented classes. In contrast, the China-Fundus-CIMT dataset exhibits less pronounced class imbalance, but presents a different problem due to the nature of the CIMT variable itself. Since CIMT values fall along a continuous spectrum, the two CIMT-based cardiovascular risk classes are defined only by a specific threshold. As will be elaborated in Section 3.3, this thresholding introduces a boundary zone ambiguity, as subjects with CIMT values near the threshold are inherently harder to classify, while those further from it are more easily distinguished. Despite the two datasets being bottlenecked by different underlying challenges, focal loss adapts to mitigate the severe class imbalance in APTOS-2019 while potentially directing greater attention to the difficult samples in China-Fundus-CIMT, particularly those with CIMT values near the boundary threshold.

It is worth noting that the class weighting in focal loss and the weighted sampling strategy in Section 2.4 tackle class imbalance at two distinct stages of the training workflow. Weighted sampling operates only at the data level to ensure minority classes are sufficiently represented within each training epoch, while the weighting factor in focal loss works at the optimization stage to further regulate how strongly these minority samples shape the gradient signal and drive parameter updates. These complementary strategies ultimately provide a much stronger mitigation of class imbalance than either could achieve alone, improving the model’s sensitivity to the underrepresented classes in the population.

### 2.6. Programming Environment and Hardware Configuration

The proposed framework was implemented in Python 3.13 using PyTorch 2.12 as the primary deep learning framework. TorchVision 0.27 was employed for image preprocessing and augmentation, while timm 1.0.27 was used to construct and implement the EfficientNet backbone. Additional Python libraries, including NumPy, pandas, and scikit-learn, were used for numerical operations, data organization, and evaluation metric computation.

All model training and evaluation experiments were performed on an NVIDIA GeForce RTX 5090 GPU with 32 GB of memory. GPU accelerated computation was performed using the NVIDIA CUDA 13.4.2 toolkit. The proposed model architectures, focal loss function, balanced sampling strategy, optimization procedures, and learning rate scheduling were all implemented within the PyTorch framework.

### 2.7. Experimental Methodology and Setup

As described in Section 2.2, the training pipeline comprises two consecutive stages: pretraining on APTOS-2019 and fine-tuning on China-Fundus-CIMT. A progressive unfreezing strategy with discriminative learning rates [37] was employed in both stages to preserve the transferable representations of the shallow layers in the pretrained backbone while allowing the deeper and more task-specific features to adapt first. All models were optimized using AdamW with a weight decay of $1\times 10^{-4}$. Since model training inherently involves stochastic processes, performance varies across runs even under the same experimental settings. To assess the stability and consistency of model performance under stochastic variations in model initialization and training, each experiment was repeated across 10 independently seeded runs using seeds 0–9. For each run, the corresponding seed was applied consistently to the relevant sources of randomness, including Python’s built-in random module and PyTorch through torch.manual_seed() and torch.cuda.manual_seed_all(). Deterministic cuDNN behavior was enforced by setting torch.backends.cudnn.deterministic = True and torch.backends.cudnn.benchmark = False. A torch.Generator initialized with the same seed was also supplied to the data loaders to ensure reproducible data loading within each run.

#### 2.7.1. Pretraining on APTOS-2019

The CBAM-enhanced EfficientNet-B4 backbone was first pretrained on the APTOS-2019 dataset. Rather than simplifying this stage to a binary distinction between the presence and absence of DR, the original five-class DR severity labels were retained to expose the network to a broader spectrum of retinal abnormalities and encourage the model to learn richer retinal representations before being transferred to the downstream task.

To construct the model, a CBAM module was first inserted between blocks 4 and 5 of the EfficientNet-B4 backbone. The EfficientNet-B4 parameters were then initialized using the ImageNet weights [26], whereas the CBAM parameters were randomly initialized. Dropout was activated at two positions, one after the CBAM module with a dropout rate of 0.3, and the other one in the classifier after the global average pooling with a dropout rate of 0.5, to mitigate the risk of overfitting [38].

Pretraining was conducted in three consecutive stages. During the first stage, blocks 0 to 4 of EfficientNet-B4 were frozen, and optimization was restricted to the CBAM module, the deeper layers, and the classifier. These components were trained for 20 epochs with an initial learning rate of $5\times 10^{-4}$, while a CosineAnnealingWarmRestarts scheduler was implemented with $T_{0}=20$, $T_{\mathrm{mult}}=1$, and $\eta_{\min}=1\times 10^{-6}$. Checkpoint with the highest validation macro-F1 was retained.

Before commencing the second stage, the best checkpoint from the preceding stage was restored and block 4 was unfrozen. A discriminative learning rate of $1\times 10^{-4}$ was assigned to the newly unfrozen block, allowing it to adapt more conservatively than the deeper and more task-specific layers. Training was continued for a further 30 epochs. The same scheduler was configured with $T_{0}=30$, $T_{\mathrm{mult}}=1$, and $\eta_{\min}=1\times 10^{-6}$. The best checkpoint was updated only when a higher F1 score was obtained. Otherwise, the previously retained checkpoint was preserved.

**Figure 2:**
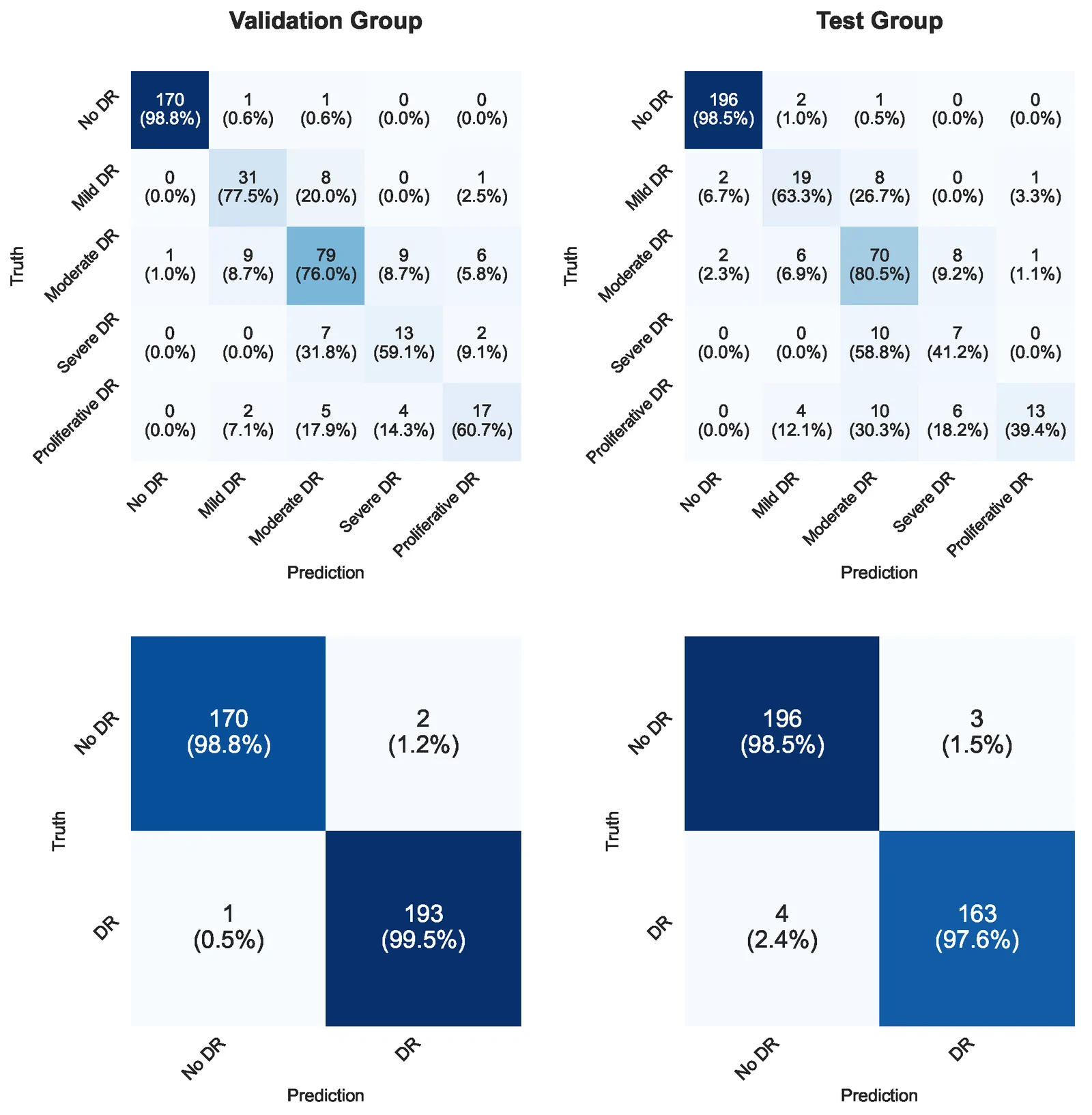
Confusion matrices for the APTOS-2019 pretrained checkpoint selected for backbone initialization in all subsequent experiments. Top row shows the five-class DR severity grading (No DR, Mild, Moderate, Severe, Proliferative) on the validation (**left**) and test (**right**) sets. Bottom row shows the same predictions collapsed into a binary No DR vs. DR grouping, demonstrating that the model reliably separates pathological from non-pathological fundus images.

In the final stage of pretraining, the best checkpoint was restored again, block 3 was unfrozen, and an even smaller learning rate of $1\times 10^{-5}$ was assigned to this block. The model was trained for another 30 epochs with the scheduler configured as $T_{0}=30$, $T_{\mathrm{mult}}=1$, and $\eta_{\min}=1\times 10^{-7}$. Subsequently, the particular checkpoint with the highest validation macro-F1 of 73.68%, whose classification performance is demonstrated in Figure 2, was used to initialize the backbones of the BMA-Net architecture in all subsequent experiments that required initialization with retinal domain pretrained weights.

It is worth noting that throughout pretraining, blocks 0 to 2 were kept frozen deliberately, a batch size of 48 was used, and an argmax decision rule was applied. Early layers of ImageNet pretrained CNNs generally encode comparatively generic visual primitives that are highly transferable to the retinal fundus domain [34]. Therefore, model adaptation was concentrated in the intermediate and deeper layers with the early representations in blocks 0 to 2 being retained.

#### 2.7.2. Fine-Tuning on China-Fundus-CIMT

As described in Section 2.2, the selected checkpoint was transferred to both backbones of the BMA-Net architecture (Figure 1b) to initialize layers from block 0 to CBAM, whereas the remaining blocks 5 and 6, MLP, and the classifier were randomly initialized. A progressive unfreezing strategy was again employed, while substantially shorter training periods and more conservative learning rates were adopted as compared to pretraining. As the fine-tuning was performed within the common retinal fundus domain, a ReduceLROnPlateau scheduler was employed to reduce the learning rate when the validation macro-F1 ceased to improve, which prevents excessive parameter updates and reduces the overfitting risks. Further regularization was applied through multiple dropout layers, one with a rate of 0.3 after the CBAM module in both backbones, and another with a rate of 0.5 between the concatenated feature representation and the MLP. A batch size of 20 was used, and an argmax decision rule was applied throughout the fine-tuning process.

Fine-tuning was performed in three stages. Initially, blocks 0 to 4 in both branches were frozen. Discriminative learning rates were assigned to the trainable layers according to their degrees of task specificity. The classifier was trained with a learning rate of $5\times 10^{-4}$, blocks 5 and 6 with $1\times 10^{-4}$, and the CBAM modules with $5\times 10^{-5}$. ReduceLROnPlateau monitored validation macro-F1 in max mode, with a reduction factor of 0.5, patience of 3 epochs, and a minimum learning rate of $1\times 10^{-5}$. This stage was limited to five epochs, and the checkpoint yielding the highest validation macro-F1 was retained.

The selected checkpoint was subsequently restored before the second fine-tuning stage, in which blocks 3 and 4 of both branches were unfrozen. A lower learning rate of $1\times 10^{-5}$ was assigned to these newly trainable blocks for more conservative adaptation of mid-level representations. The model was trained for another 10 epochs, with the minimum learning rate of the ReduceLROnPlateau scheduler set to $1\times 10^{-6}$ and all other parameters unchanged. Checkpoint selection was determined by the highest validation macro-F1.

In the final stage, the previous best checkpoint was restored, and all remaining blocks were unfrozen to enable end-to-end fine-tuning of the network. These newly unfrozen early layers were assigned the smallest learning rate of $1\times 10^{-6}$, making only limited adjustments to the most generic and transferable pretrained representations. The scheduler retained the same settings, except that the minimum learning rate was further reduced to $1\times 10^{-7}$. The fully trainable network was optimized for a final 10 epochs, with the checkpoint achieving the highest validation macro-F1 selected as the final model.

#### 2.7.3. Evaluation Metrics

Model performance was evaluated using the macro-averaged F1 score, class-wise F1 scores [39], area under the receiver operating characteristic curve (AUROC), and area under the precision-recall curve (AUPRC) [40]. These metrics provide complementary assessments of classification performance and account for class imbalance in the dataset.

Macro-F1 assigns equal weight to each class regardless of class frequency and was therefore selected as the primary metric for assessing overall balanced classification. Class-wise F1 scores were used to evaluate the normal and thickened classes separately by balancing precision and recall within each class. Complementing these F1-based measures, AUROC assesses the model’s ability to discriminate between the normal and thickened classes across classification thresholds, while AUPRC summarizes the trade-off between precision and recall and is especially informative in the presence of class imbalance [41]. In particular, AUPRC was estimated using average precision (average_precision_score() from scikit-learn), with the predicted probabilities for the CIMT-thickened class used as the prediction scores.

### 2.8. Ablation Studies

A series of ablation experiments was conducted to qualitatively and quantitatively evaluate the contributions of key components within the proposed training framework. Each experiment was designed to isolate a specific transfer learning choice and assess its effect on downstream CIMT-based cardiovascular risk classification. In particular, the experiments examined whether transferring CBAM parameters pretrained in DR domain provided a favorable initialization for CIMT-based classification, whether progressive adaptation of the shallow backbone layers improved generalization, and whether the new training pipeline offered an advantage over conventional ImageNet transfer learning.

For consistency, the model configuration and regularization settings were kept fixed unless explicitly stated otherwise. Throughout this section, the shallow layers of the backbone refer to the convolutional stem and blocks 0 to 4 of each branch. Dropout was applied at two positions of the architecture, one after the CBAM module in each backbone, with a dropout rate of 0.3, and the other one after the concatenated feature representation immediately before the MLP, with a dropout rate of 0.5. A weight decay of $1\;\times 10^{-4}$ was applied consistently across all experiments. For each ablation configuration, experiments were repeated across 10 different random seeds. For each seeded training run, the model checkpoint achieving the highest validation macro-F1 score was selected for further evaluation. Moreover, ablation experiments 1–3 used a batch size of 20, whereas the final ablation experiment used a batch size of 16. All ablation experiments applied an argmax decision rule. For ease of reference, the training configurations and hyperparameters used for the main configuration and all the ablation experiments are summarized in Table 1.

#### 2.8.1. Ablation 1: Reinitialized CBAM, Progressive Unfreezing of Shallow Layers

This ablation study investigated how the pretrained CBAM affected downstream classification performance. Specifically, it evaluated whether transferring CBAM parameters learned in the DR domain provided a more effective initialization than reinitializing the attention modules from scratch.

To isolate this effect, the pretrained weights for the shallow layers of the backbone were retained for both branches, while the CBAM modules were reinitialized using Kaiming initialization. All other configurations of the training pipeline were kept identical to Section 2.7.2, including the progressive unfreezing strategy, discriminative learning rates, unfreezing schedule, total number of epochs, learning-rate scheduler, and model selection.

#### 2.8.2. Ablation 2: Pretrained CBAM, Shallow Layers Frozen

This ablation was designed to evaluate the contribution of the progressive unfreezing training strategy to the downstream classification performance. Specifically, it examined whether fine-tuning the shallow backbone layers improved generalization beyond the representations acquired during the retinal domain pretraining, thereby determining the extent to which these features required further task-specific adaptation.

To this end, the shallow layers of the backbone were initialized using the APTOS-2019 pretrained weights and kept frozen throughout training to retain the full benefit of retinal domain pretraining. The pretrained CBAM modules, together with blocks 5 and 6, MLP, and the classifier, were left trainable. Discriminative learning rates were applied to the trainable modules, with the classifier at $1\times 10^{-4}$, blocks 5 and 6 at $5\times 10^{-5}$, and the pretrained CBAM modules at $1\times 10^{-5}$. The ReduceLROnPlateau scheduler was configured with a reduction factor of 0.5 and a minimum learning rate of $1\times 10^{-6}$. Training was conducted for 30 epochs, and the checkpoint with the highest validation F1 was selected for further evaluation.

#### 2.8.3. Ablation 3: Reinitialized CBAM, Shallow Layers Frozen

This ablation was designed to assess the effectiveness of CBAM pretraining when the early backbone layers remained frozen. Specifically, it examined whether the transferred CBAM parameters provided a performance benefit without further adaptation of these layers, or whether such a benefit was only observed when progressive unfreezing was applied.

In this setting, the shallow backbone layers were again initialized from the APTOS-2019 pretrained checkpoint and kept frozen throughout training. However, the CBAM modules in both branches were reinitialized using Kaiming initialization instead of retaining the pretrained parameters. The remaining layers including the reinitialized CBAM modules were left trainable. All the optimization configurations were kept identical to the second ablation to ensure a fair comparison.

#### 2.8.4. Ablation 4: Reinitialized CBAM, ImageNet Backbone

This ablation study compared the retinal domain and ImageNet-based transfer learning pipelines for downstream CIMT-based cardiovascular risk classification. Specifically, the EfficientNet backbones in both branches were initialized using ImageNet weights rather than the weights from APTOS-2019 pretraining. Since the ImageNet model did not contain pretrained CBAM parameters, the CBAM modules in both branches were initialized using Kaiming initialization to be consistent with the preceding ablations, while the MLP and classifier were initialized using the default scheme.

The effectiveness of transfer learning strategies, such as progressive unfreezing and discriminative learning rates, can be limited by the substantial differences between the source and target domains [42]. For this ablation, ImageNet weights were adopted which originated from the natural image domain, resulting in a significant domain shift relative to the target retinal images. Therefore, rather than applying the same progressive unfreezing strategy used in the preceding ablations, the entire architecture was made trainable from the outset to allow greater flexibility in adapting the ImageNet representations to the downstream CIMT-based cardiovascular risk classification task. The optimization strategy was also adjusted accordingly. For the first 20 epochs, the optimizer was configured with an initial learning rate of $1\times 10^{-4}$, and a StepLR scheduler was used to decay this learning rate by a factor of γ=0.5 every 5 epochs. The optimizer was then reinitialized with a lower learning rate of $5\times 10^{-5}$, and training was continued for another 10 epochs under the same StepLR configuration.

Given the differences in optimization and fine-tuning strategies, this ablation should be interpreted as a comparison of differently optimized transfer learning pipelines rather than a controlled test isolating the effect of retinal domain versus ImageNet initialization.

## 3. Experimental Analysis and Results

### 3.1. Evaluation of Pretraining on APTOS-2019

Following the training scheme described in Section 2.7.1, the best checkpoint was obtained, achieving a validation macro-F1 of 73.68%. When evaluated on the test set using the original five-class DR grading, the model achieved a macro-F1 of 65.36%. The class specific performance on the validation and test sets is further illustrated by the top two confusion matrices in Figure 2. Although the model achieved only moderate performance in fine-grained five-class DR grading, this did not necessarily indicate that the model is not suitable for transfer learning to the downstream task. Yan et al. [43] observed that the major difficulty of DR classification lies in distinguishing the subtle differences among individual DR grades, which requires the model to be able to extract highly DR grade-specific features. Therefore, the moderate performance may only reflect an insufficient representation of these grade-specific features rather than a lack of relevant knowledge in the retinal domain. Moreover, features that are highly specific to distinguishing between individual DR grades offer limited benefit for the downstream task and could potentially reduce transferability if the model becomes overly specialized to the pretraining objective [34].

To assess whether the pretrained model had acquired general retinal representations despite its moderate performance in fine-grained five-class DR grading, DR grades 1 to 4 were grouped into a single DR class and evaluated against the non-DR class. As shown in the lower two confusion matrices in Figure 2, performance improved substantially under this binary formulation, achieving macro-F1 scores of 99.18% and 98.07% on the validation and test sets respectively. These results suggest that although the model was less effective at distinguishing the severity of DR, it was highly capable of differentiating pathological from non-pathological retinal images, demonstrating that it had learned relevant retinal domain representations.

### 3.2. Evaluation of Fine-Tuning on China-Fundus-CIMT

**Figure 3:**
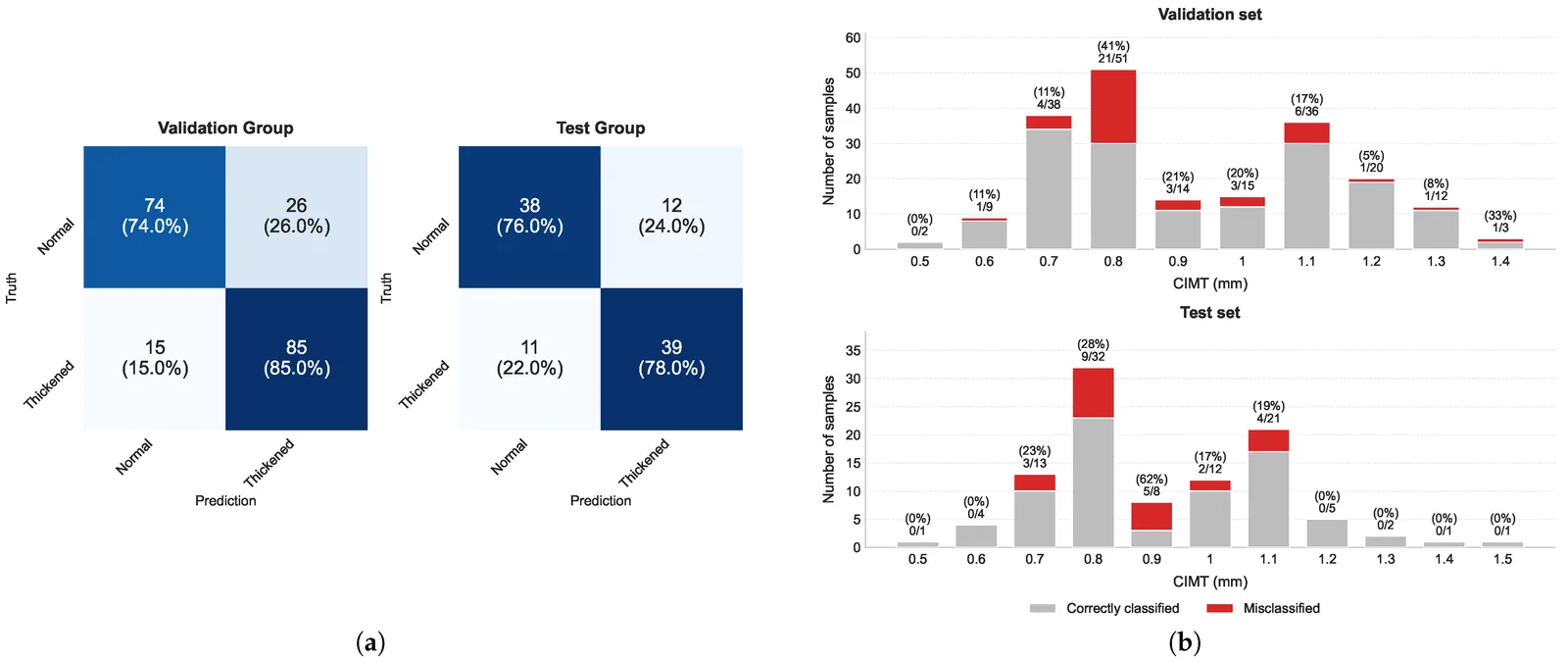
Classification performance of the representative BMA-Net model (Single Seed) on the China-Fundus-CIMT validation and test sets. (**a**) Confusion matrices for the normal and thickened classes on the validation (left) and test (right) sets. (**b**) Number and proportion of correctly classified (grey) and misclassified (red) samples by the BMA-Net (Single Seed) model, as grouped by ground-truth CIMT value (mm), for the validation (top) and test (bottom) sets. Misclassifications are concentrated within the 0.7–1.1 mm range around the classification boundary.

Ten independently trained BMA-Net models were obtained from different random initializations using the procedure described in Section 2.7.2. For each model, performance was evaluated using the class-wise and macro-averaged F1 scores, AUROC, and AUPRC on both the validation and test sets. These metrics were subsequently averaged across the ten seeded runs to characterize the overall performance of models under the proposed training scheme and configuration. Specifically, in Table 2, the row denoted as BMA-Net (Single Seed) corresponds to a run that exhibited consistently strong performance across all the reported metrics while maintaining comparable performance between the validation and test sets. Moreover, the classification outcomes of the selected run on both the validation and test sets are visualized through the confusion matrices presented in Figure 3a. Although this run did not achieve the best performance on any individual metric, it was selected as a representative example of the performance obtained under this training scheme, rather than as a best-case result. In comparison, the Siamese SE-ResNeXt row reports classification using the architecture and training scheme proposed in [16]. As AUPRC was not reported in the original study, no corresponding baseline value is included in Table 2.

The selected run of the BMA-Net achieved macro-F1 scores of 79.44%/77.00% and AUROC values of 83.24%/85.52% on the validation and test sets, while the Siamese SE-ResNeXt model achieved macro-F1 scores of 77.20%/74.94% and AUROC values of 82.79%/82.58% respectively. Compared with previous Siamese SE-ResNeXt results shown in Table 2, BMA-Net achieved relative increases of macro-F1 scores by 2.90% and 2.75%, and AUROC by 0.54% and 3.56% on the validation and test sets, respectively. Beyond the differences in aggregate performance, the proposed configuration also effectively reduced the disparity in classification performance between the normal and thickened classes, as reflected by the class-wise F1 scores. Under the original Siamese SE-ResNeXt configuration, the absolute differences between the class-wise F1 scores were 5.24 percentage points (pp) on the validation set and 2.51 pp on the test set. For the selected BMA-Net model, these differences were reduced to 2.26 pp and 0.46 pp respectively. These reduced disparities further indicate greater robustness of the proposed architecture and training scheme to the class imbalance present in the dataset. The Siamese SE-ResNeXt results are directly taken from the original study [16], so they serve primarily as a reference benchmark for the present analysis.

The performance metrics were averaged across ten seeded runs and reported as BMA-Net (Average) in Table 2. Notably, these metrics were broadly consistent with those obtained from the selected run. This further indicates that the performance reported in the preceding paragraphs for the selected run reflects a pattern that is broadly reproducible under the proposed training configuration.

Currently, there is limited state-of-the-art research on CIMT-based cardiovascular risk classification from bilateral fundus images. The preceding work by Gong et al. [15] already conducted a comprehensive architectural comparison for the same underlying task and identified the Siamese formulation as the strongest overall configuration. The subsequent Siamese SE-ResNeXt model reported by Guo et al. [16] therefore provides the most relevant published benchmark on the China-Fundus-CIMT dataset.

### 3.3. Analysis of Validation and Test Result Patterns on China-Fundus-CIMT

As shown by the confusion matrices of the selected run in Figure 3a, the thickened group was classified more accurately than the normal group on the validation set. However, this performance gap was considerably less pronounced on the test set. Moreover, a related discrepancy between the validation and test performance was observed across several independently seeded runs, in which the test macro-F1 exceeded the validation macro-F1 by a notable margin. This behavior is somewhat unexpected, as the test performance would be expected to be comparable to or somewhat lower than the validation performance, since the validation performance was used to guide checkpoint selection, while the test set remained entirely unseen during model development [44]. Therefore, the repeated occurrence of notably higher test macro-F1 scores warrants further investigation, particularly as the strict separation of the training, validation, and test partitions rules out data leakage as a plausible explanation. In addition, although sampling variability means that test performance can exceed validation performance in an individual run, its repeated occurrence across multiple random initializations, as well as the reduced class-wise performance disparity observed on the test set, suggest that the difference may reflect systematic variation in sample difficulty between the two partitions rather than random fluctuation alone.

**Figure 4:**
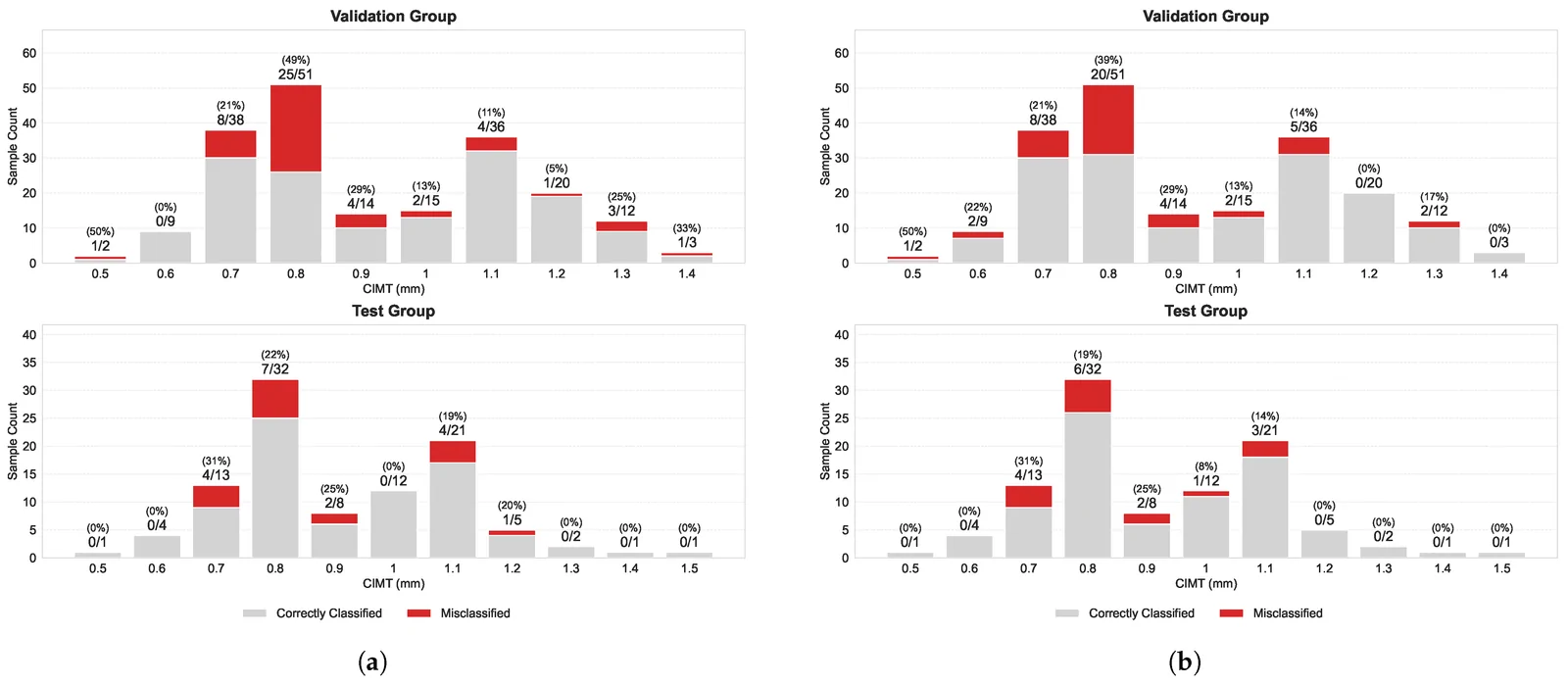
Distribution of correctly classified (grey) and misclassified (red) samples according to CIMT thickness for two additional independently seeded BMA-Net checkpoints for which test macro-F1 exceeded validation macro-F1. (**a**) Checkpoint with validation/test macro-F1 scores of 75.28%/81.97%. (**b**) Checkpoint with validation/test macro-F1 scores of 77.82%/83.97%. For each panel, the validation and test results are shown at the top and bottom, respectively. Consistent with Figure 3b, misclassifications are concentrated near the classification boundary, with a consistent drop in error rate at 0.8 mm between the validation and test sets.

To further investigate this discrepancy, two additional checkpoints were selected for which the test macro-F1 exceeded the validation macro-F1. Specifically, one checkpoint achieved macro-F1 scores of 75.28% and 81.97% on the validation and test sets, while the other achieved 77.82% and 83.97%. Subsequently, the number and proportion of misclassified samples for these two checkpoints, along with the originally selected model, were then aggregated by CIMT value and are presented as bar charts in Figure 3b and Figure 4 respectively. Across the three bar charts, misclassifications were all concentrated around the classification boundary, particularly within the CIMT range of 0.7 to 1.1 mm. Notably, a consistent pattern emerged at 0.8 mm, where the largest reduction in misclassification rate between the validation and test sets was observed for all three checkpoints. This consistent drop in errors for the 0.8 mm subgroup suggests that these boundary cases may be inherently more difficult to be classified within the validation partition than the test partition under the current training scheme, which offers a plausible explanation for the unexpected model performance on the test group. However, this is a *post hoc* analysis and whether it reflects a more general and systematic issue in the dataset or the population warrants further investigation.

### 3.4. Evaluation of Ablation Results

**Figure 5:**
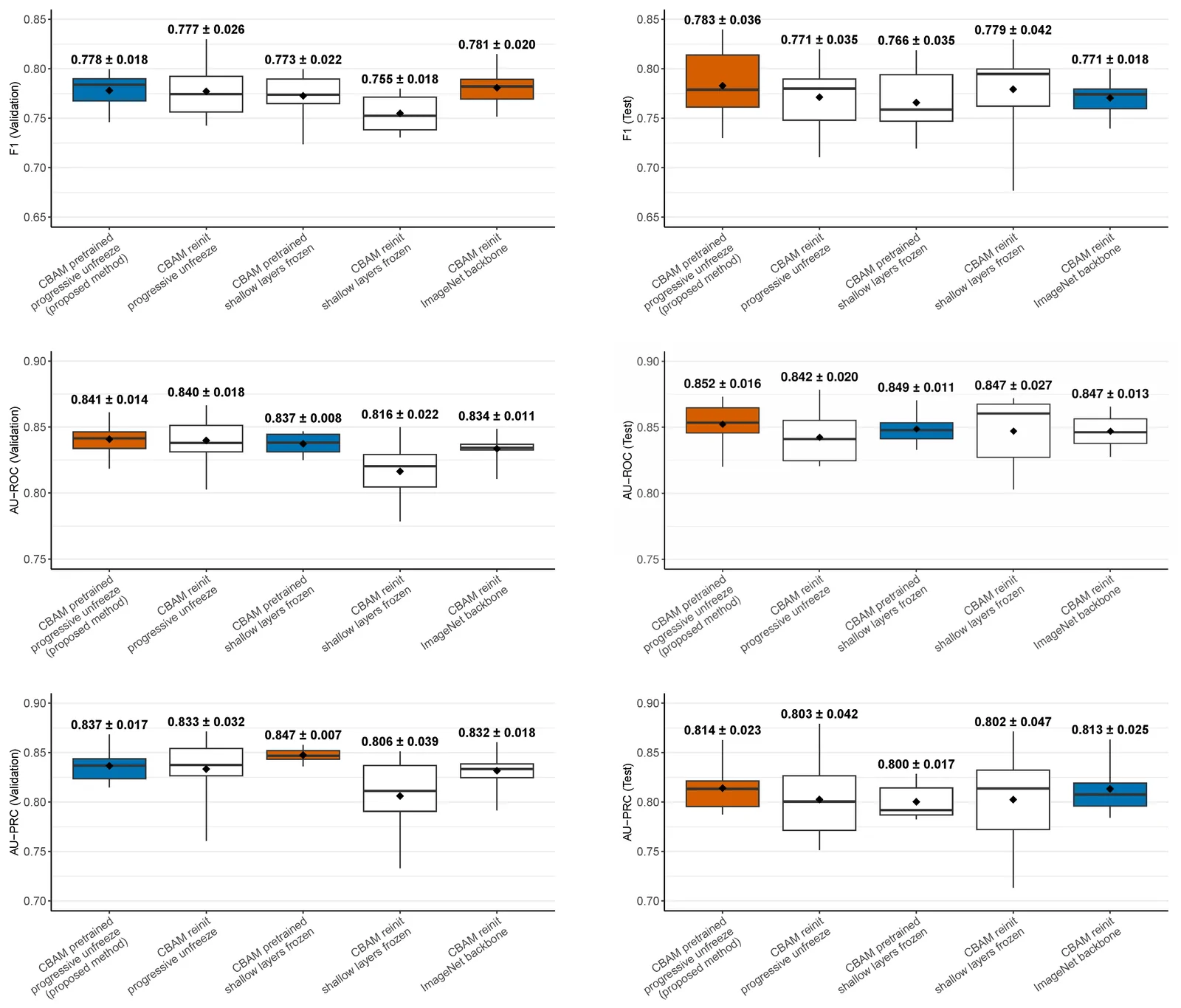
Distributions of macro-F1 (**top**), AUROC (**middle**), and AUPRC (**bottom**) across ten independently seeded runs for each of the five training configurations examined in the ablation study (Section 2.8), evaluated on the validation (**left**) and test (**right**) sets. CBAM pretrained indicates the initialization of the CBAM modules using weights learned during pretraining, whereas CBAM reinit indicates the reinitialization of the CBAM modules before fine-tuning. Progressive unfreezing refers to the sequential unfreezing of backbone layers during fine-tuning, whereas shallow layers frozen indicates that the convolutional stem and blocks 0 to 4 remain frozen during fine-tuning. The second through fifth configurations (from left to right) correspond to Ablations 1–4, respectively. Their configurations are summarized in Table 1 and described in detail in Section 2.8. Mean ± standard deviation is reported above each box; the best and second-best performing configuration for each metric and partition are highlighted in orange and blue, respectively.

Ablation experiments were conducted to assess the contributions of the proposed pretraining and fine-tuning strategies to the classification performance and stability of training across independent random initializations. For each training configuration described in Section 2.8, ten independent runs were performed, with macro-F1, AUROC, and AUPRC evaluated on both the validation and test sets. It should be noted that, as both the validation and test sets are balanced, the AUPRC baseline, determined by the prevalence of the positive class, is 0.5 for both sets. Accordingly, the resulting distributions of model performance, represented by the values of the different metrics across the ten runs, are presented in Figure 5, allowing both the central tendency and run-to-run variability of each configuration to be examined. The mean and standard deviation are additionally reported above each boxplot to summarize the average predictive performance and the sensitivity of each training configuration to random initialization. In each subplot of Figure 5, the five configurations were compared primarily based on their means and medians. The run-to-run variability was considered as a secondary criterion and was assessed based on the spread of the boxplots and the standard deviations. In general, configurations with higher means and medians also exhibited relatively low variability. In cases where a configuration with higher mean and median exhibited greater variability than another configuration, the configuration with higher mean and median was preferred unless the increase in variability was substantial while the differences in means and medians were relatively small. The best and second-best configurations were therefore identified through this qualitative comparison and highlighted in orange and blue respectively.

Overall, the proposed configuration, which combines APTOS-2019 pretraining with progressive unfreezing, demonstrated the strongest and most consistent performance across all metrics on the validation and test sets. It achieved consistently high means and medians for the reported metrics while exhibiting comparatively low variability across independent runs. By contrast, the training configuration that reinitialized CBAM while keeping the shallow pretrained layers frozen resulted in the poorest overall performance. Moreover, the ablation results in Figure 5 reveal a progressive deterioration in both predictive performance and training stability across random initializations as the pretrained CBAM parameters and progressive unfreezing of the shallow layers are removed. This suggests that the proposed transfer learning configuration improves the robustness of the training process to variations in random initialization.

A further notable finding was the comparatively strong numerical performance achieved with ImageNet weights. Despite the absence of retinal domain pretraining, this configuration remained competitive with those initialized with APTOS-2019 pretrained weights and, for some metrics, achieved comparable or superior results. In particular, it achieved the highest validation macro-F1 amongst all the configurations. However, similar predictive performance does not necessarily imply that models rely on the same image features or learn equally relevant and useful representations [45]. This observation therefore motivated a subsequent Grad-CAM analysis to qualitatively examine the differences in spatial regions contributing to the model predictions exhibited by the ImageNet-based transfer learning and the proposed training pipeline.

### 3.5. Analysis of Spatial Attention via Grad-CAM

Grad-CAM was implemented to compare the spatial attention patterns learned under different training strategies. Specifically, checkpoints from three training configurations were selected for comparison, which include the proposed method, the ImageNet pretrained model, and the lowest performing configuration in which the CBAM parameters were reinitialized and progressive unfreezing was not applied. We additionally selected four patients to illustrate the common attention patterns observed across these examined samples. These comprised one easy and one challenging example from each class, as determined by the models’ prediction confidence. As Grad-CAM provides only qualitative visualization, these patterns are not necessarily consistent across all samples.

**Figure 6:**
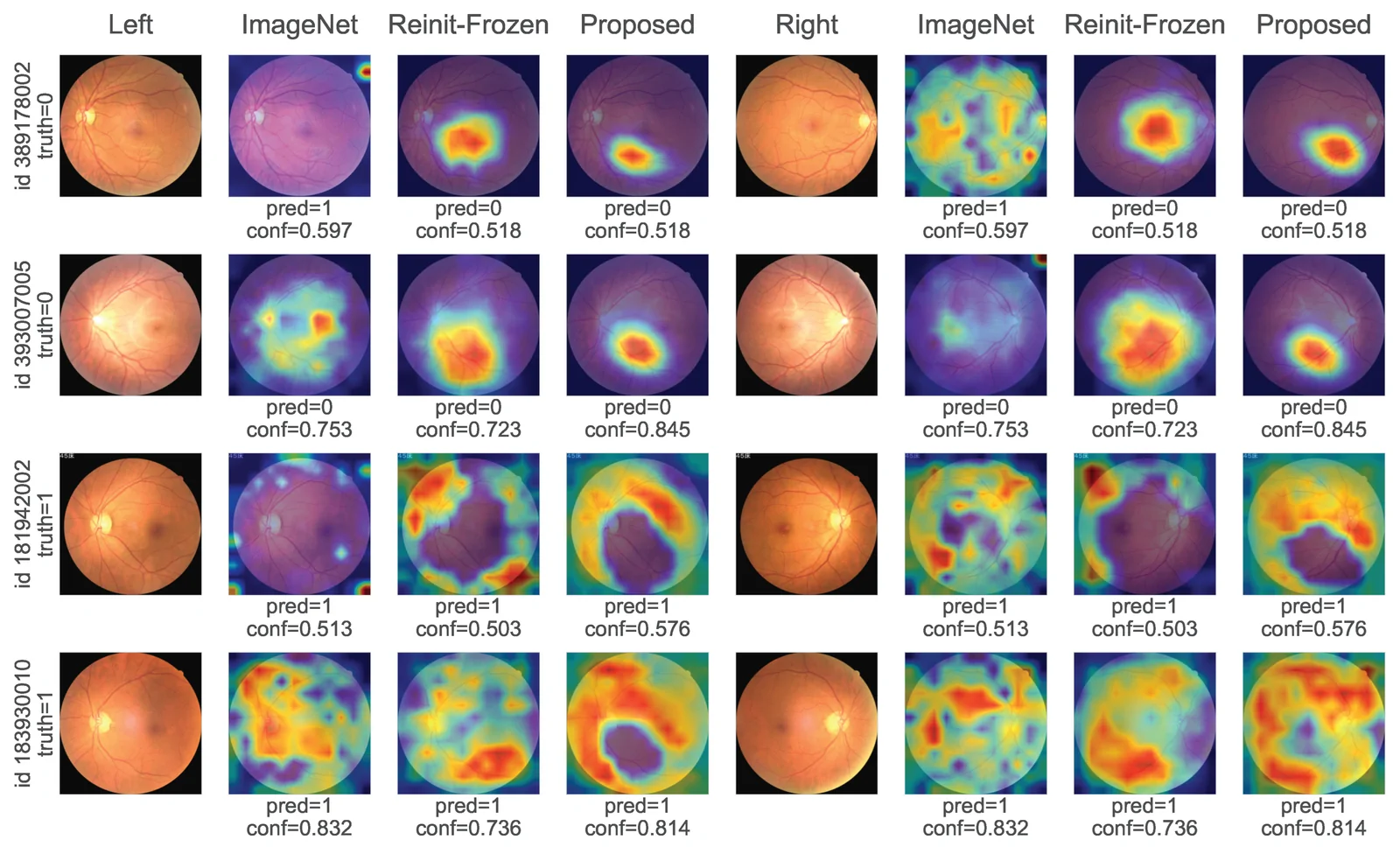
Grad-CAM visualizations for four representative subjects comparing spatial activation patterns from the ImageNet-pretrained backbone, the reinitialized CBAM configuration trained with frozen shallow layers (lowest-performing ablation), and the proposed method. Rows show one easy and one challenging example from each class (ID 389178002 and 393007005: normal; ID 181942002 and 183930010: thickened), with Grad-CAM overlays for the left and right eyes displayed side by side along each row. Predicted class and prediction confidence are annotated below each panel.

As shown in Figure 6, the proposed model produced relatively consistent activation patterns across both the selected easy and difficult samples, with the spatial distribution of activation patterns varying according to the predicted class. For samples classified as thickened, activation was distributed predominantly along the entire retinal vasculature. This pattern remained apparent even in difficult samples, for which the confidence was only slightly above 0.5. In contrast, samples classified as normal exhibited more concentrated activation around the inferotemporal vascular arcade.

The checkpoint from the lowest performing training configuration also produced activation patterns that were largely localized to retinal structures. However, for the difficult samples, regions with strong activation patterns showed some deviation from those in the easy samples, while the overall activation remained broadly consistent. As the differences mainly involved shifts in the location and extent of the strongest activation rather than activation in anatomically unrelated regions, the model appeared to retain the anatomically meaningful attention patterns across these samples.

Despite the comparatively strong classification performance achieved by the ImageNet pretrained model, its activation maps demonstrated substantial variations in spatial attention across the selected examples, particularly for the difficult ones. In several cases, strong activation was concentrated predominantly in only one of the bilateral fundus images, while the corresponding retinal regions in the other image exhibited weak or negligible activation. Pronounced off-target focal activation was also observed within the black background surrounding the fundus images.

## 4. Discussion

The results suggest that the proposed training strategy improves not only the model’s predictive ability but also its robustness to random initializations. The reduced gap in class-wise F1 further indicates that the proposed training strategies may alleviate the original model’s bias towards the thickened class. Beyond these quantitative improvements, the Grad-CAM analysis provides complementary insights into model behavior that are not captured by the classification metrics. Despite the competitive numerical performance of the ImageNet pretrained model, its activation patterns exhibit significant variations across the selected easy and difficult samples. This observed inconsistency in its activation behavior highlights the need for further systematic analysis before drawing conclusions regarding its reliability.

Moreover, DR domain pretraining with APTOS-2019 is particularly valuable given the limited amount of task-specific data available for CIMT-based cardiovascular risk classification. Instead of learning early retinal representations solely from the available CIMT-labelled fundus images, the model can be first exposed to a larger retinal image dataset and then fine-tuned to the target task. The anatomically localized activation patterns observed after fine-tuning suggest that features acquired during pretraining remain largely relevant following this adaptation. However, these qualitative findings should be interpreted with caution. Grad-CAM highlights image regions that contribute to the model’s prediction, but these attribution maps reflect the model’s learned decision process rather than establishing a biological or causal relationship between the highlighted retinal structures and CIMT-based cardiovascular risk [22,46].

Another point worth revisiting is the concentration of classification errors around the CIMT decision boundary discussed in Section 3.3. The binary labels impose a discrete separation between normal (CIMT<0.9 mm) and thickened (CIMT≥0.9 mm) cases, despite the underlying physiological measurement varying continuously. However, there is no reason to assume that retinal manifestations associated with CIMT-based cardiovascular risk change abruptly between CIMT values of 0.8 and 0.9 mm. Cases near this boundary may actually be less visually separable than their binary labels suggest, while small differences in CIMT may also be influenced by individual physiological variation and measurement uncertainty. Prior work [47,48] demonstrates that discretizing continuous variables, including biomarkers, at an arbitrary threshold introduces discretization noise, as observations near the cut-off may have similar underlying physiological characteristics despite being assigned to different classes. Therefore, the concentration of errors observed around the boundary reflects, at least in part, the ambiguity in the formulation of the prediction task rather than simply the inability of the model to distinguish the two classes.

This observation motivates the consideration of alternative formulations that better accommodate uncertainty around intermediate CIMT values. Future work could, for example, investigate an intermediate-risk category, an ordinal or continuous prediction framework, or a learning-to-defer approach in which the model is permitted to defer uncertain cases for further assessment. Such formulations may better reflect the continuous nature of CIMT and avoid forcing borderline cases into sharply separated risk categories, although their clinical validity would need to be established independently.

In addition to the limitations discussed above, the relatively small and imbalanced China-Fundus-CIMT dataset may constrain the generalizability of the proposed framework. The absence of external validation on an independent cohort further limits the extent to which the present findings can be generalized beyond the current dataset. Additionally, the present study only characterizes variability arising from stochastic model training across random seeds and does not quantify statistical uncertainty at the patient level. In particular, patient-level confidence intervals for the performance metrics were not estimated. Future work could address this limitation by estimating the confidence intervals of the metrics using subject-level resampling methods.

## 5. Conclusions

This study presents the BMA-Net architecture and its associated retinal domain transfer learning strategy for CIMT-based cardiovascular risk classification of bilateral fundus images. By combining APTOS-2019 pretraining with progressive model adaptation, the proposed approach achieved strong classification performance with reduced variability across random initializations, while also narrowing the difference in classification performance between the normal and thickened classes. Furthermore, Grad-CAM analysis also illustrated that the proposed training pipeline produced consistent activation patterns. These findings support the potential value of DR domain pretraining for CIMT-based classification, particularly when task-specific training data are limited. Moreover, an important direction for future work is to assess whether the benefits of retinal domain pretraining extend across different populations, imaging devices, and clinical settings. Such evaluation is currently constrained by the limited availability of independent fundus datasets with comparable CIMT annotations and class definitions [16]. As suitable datasets become available, external validation will be valuable for establishing whether the observed improvements are maintained across independent cohorts and acquisition settings.

## Figures and Tables

**Table 1:** Summary of training configurations and hyperparameters for experiments on the China-Fundus-CIMT dataset.

Hyperparameter	BMA-Net	Ablation 1	Ablation 2	Ablation 3	Ablation 4
Backbone initialization	APTOS-2019	APTOS-2019	APTOS-2019	APTOS-2019	ImageNet
CBAM initialization	Pretrained	Kaiming	Pretrained	Kaiming	Kaiming
Shallow layers	Progressive unfreezing	Progressive unfreezing	Frozen	Frozen	Trainable from outset
LR strategy	Discriminative	Discriminative	Discriminative	Discriminative	Two-stage global
Classifier LR	$5\times 10^{-4}$	$5\times 10^{-4}$	$1\times 10^{-4}$	$1\times 10^{-4}$	$1\times 10^{-4}$/$5\times 10^{-5}$
Blocks 5–6 LR	$1\times 10^{-4}$	$1\times 10^{-4}$	$5\times 10^{-5}$	$5\times 10^{-5}$	Global LR
CBAM LR	$5\times 10^{-5}$	$5\times 10^{-5}$	$1\times 10^{-5}$	$1\times 10^{-5}$	Global LR
LR scheduler	ReduceLROnPlateau	ReduceLROnPlateau	ReduceLROnPlateau	ReduceLROnPlateau	StepLR
Scheduler parameters	Factor =0.5; Patience =3; min LR $=1\times 10^{-5}$	Factor =0.5; Patience =3; min LR $=1\times 10^{-5}$	Factor =0.5; Patience =3; min LR $=1\times 10^{-6}$	Factor =0.5; Patience =3; min LR $=1\times 10^{-6}$	γ=0.5; Step =5
Training epochs	25 (5 + 10 + 10)	25 (5 + 10 + 10)	30	30	30 (20 + 10)
Batch size	20	20	20	20	16
Classification rule	argmax				
Dropout (CBAM/MLP)	0.3/0.5				
Weight decay	$1\times 10^{-4}$				

**Table 2:** Classification performance of BMA-Net and the baseline Siamese SE-ResNeXt [16] on the validation and test sets, reported as class-wise and macro-averaged F1, AUROC, and AUPRC. BMA-Net (Single Seed) denotes a representative run selected from ten independently initialized models; BMA-Net (Average) reports metrics averaged across all ten runs. AUPRC was not reported for the Siamese SE-ResNeXt baseline, as it was not included in the original study.

Model	Group	F1 (%)			AUROC (%)	AUPRC (%)
		Normal	Thickened	Macro		
Siamese SE-ResNeXt	Validation	74.58	79.82	77.20	82.79	–
	Test	73.68	76.19	74.94	82.58	–
BMA-Net (Single Seed)	Validation	78.31	80.57	79.44	83.24	82.63
	Test	76.77	77.23	77.00	85.52	81.89
BMA-Net (Average)	Validation	77.12	78.49	77.81	84.08	83.67
	Test	78.59	77.99	78.29	85.22	81.40

## Data Availability

The China-Fundus-CIMT dataset analyzed in this study is publicly available on Figshare at https://doi.org/10.6084/m9.figshare.27907056. The original data collection was granted an exemption from informed consent by the Ethics Committee of the Second Affiliated Hospital of Anhui Medical University (Ethics Approval No. YX2023-2011(F1)), and the released data were fully anonymized. The APTOS-2019 dataset was originally released through the APTOS 2019 Blindness Detection competition on Kaggle via https://www.kaggle.com/c/aptos2019-blindness-detection (accessed on 15 October 2025). The data and corresponding partitions used in this study were obtained from https://www.kaggle.com/datasets/mariaherrerot/aptos2019 (accessed on 15 October 2025) and were used for academic research in accordance with the applicable Kaggle competition data-use requirements. The present study involved secondary analysis of these existing datasets and did not involve new participant recruitment or collection of identifiable personal data. The code is available at https://github.com/MultiMeDIA-Oxford/BMA-Net (accessed 30 September 2026).
